# Preventive effects of *Prangos ferulacea (L.) *Lindle on liver damage of diabetic rats induced by alloxan

**Published:** 2012

**Authors:** Farah Farokhi, Najme Kaffash Farkhad, Amir Togmechi, Khosro Soltani band

**Affiliations:** 1 *Department of Biology, Faculty of Science, Urmia University, I. R. Iran*; 2 *Histology and Embryology, Department of Biology, Faculty of Science, Urmia University, I. R. Iran*; 3 *Department Artemias Rresearch, Faculty of Veterinary, Urmia University, I. R. Iran*

**Keywords:** ALT, AST, Diabetes mellitus, Histopathology

## Abstract

**Objectives: **Diabetes mellitus is associated with biochemical, physiological and pathological alterations in the liver. The aim of this study was to investigate the effects of hydroalcoholic extract of *Prangos ferulacea (L.) *Lindle (P.f) on changes in rats´ liver structure and serum activities of alanin and aspartate aminotransferases after alloxan injection.

**Materials and Methods: **In this study, forty female Wistar rats with body weight of 200±20 g were randomly divided into 5 groups with 8 rats per group. Diabetes was induced in rats by alloxan monohydrate at dose of 120 mg/kg body weight (BW) injected intraperitoneally. Root and leaves with stems hydroalcoholic extract of P.f at dose of 100 mg/kg BW were given orally in diabetic rats daily for a month.

**Results: **In diabetic rats (D) the serum allanin aminotransferases (ALT) and aspartate aminotransferases (AST) were significantly increased (p<0.05) in comparison with the other groups. Moreover, in this group, necrosis of hepatocytes, cytoplasmic vacuolations, and lymphocytic inflammation were observed. Diabetic rats treated by root extract of P.f compared with diabetic group showed a significant decrease in these enzymes. In addition, in this group all of previous signs were improved.

**Conclusion: **Root hydroalcoholic extract of P.f found to influence changes of aminotransferases and prevent the histopathological changes of liver associated with alloxan diabetes in rats.

## Introduction

Diabetes mellitus is a chronic metabolic disorder that continues to present a major worldwide health problem. It is characterized by absolute or relative deficiencies in insulin secretion and/or insulin action associated with chronic hyperglycemia and disturbances of carbohydrate, lipid, and protein metabolism (Duckworth, 2001 ▶). It is accepted that oxidative stress results from an imbalance between the generation of oxygen derived radicals and the organism antioxidant potential (Abdollahi et al., 2004 ▶). Various studies have shown that diabetes mellitus is associated with increased formation of free radicals and decrease in antioxidant potential due to these events (Nazirogilu et al., 2005 ▶). There are multiple changes in diabetic patients that are associated with oxidative stress such as: glycation of some products (Diabetes Care, 2010 ▶) and hypoxia resulting from hyperglycemia (Kaneto et al., 2007 ▶) that can make an imbalance in cellular oxidation and reduction, especially in liver tissue (Gallou et al., 2007 ▶).

Liver is a big organ that its main function is managing and controlling carbohydrates, lipids, and proteins metabolism. Maintaining normal blood glucose levels by taking and storing glucose in form of glycogen (glycogenesis), cleavage of glycogen into glucose (glycogenolysis), and forming glucose from non-carbohydrate sources such as amino acids (gluconeogenesis) are some other functions of liver (Worbozet, 2003 ▶). Various studies have shown that alloxan has deleterious effects on liver and kidney (Giannini et al.,2005 ▶). Disruption in livers function that is demonstrated with increasing in allanin and aspartate aminotransferases (ALT & AST) have been reported one week after alloxan injection (Yamatani et al., 1994 ▶; Zafar et al., 2009 ▶). These enzymes, gamma-glutamil transferases (γGT) and billirubin are measured for investigating livers function (Zafar et al., 2009 ▶). Aminotransferases are the markers of the healthy hepatocytes (Pratt et al., 2009 ▶). ALT is mainly found in the liver but AST is found in the liver and some other organs, so it is a less-specific marker for liver (Pratt et al., 2009 ▶). Liver has a major role for maintaining post-prandial normal glucose concentration and it is the main site of insulin clearance (Pratt et al., 2009 ▶). Many studies have shown an association between specific diabetic complications and disturbances in various tissues, such as diabetic nephropathy and peripheral neuropathy, but only limited data is available on the possible association between diabetic complication and liver functions.

Herbal remedies are extracted from various plants and used for treating different disease such as inflammatory diseases, diabetes mellitus, and various hepatic and gastrointestinal diseases (Vozarova et al., 2006 ▶; Silva et al., 2008 ▶). Recently, there has been a considerable interest in finding natural antioxidant from plant materials to replace synthetic ones. Data from both scientific reports and laboratory studies show that plants contain a large variety of substances that possess antioxidant activity (Shanmugasundaram et al., 2008 ▶). Phytomedicals with antioxidant effects include cinnamic acids, coumarins, diterpenes, flavonoides, monoterpenes, phenylpropanpides, and triterpenes (Srivastava et al., 2003 ▶). In this aspect, *Prangos ferulacea (L.) *Lindle (P.f) is a plant native to the mountains of southern Iran (Fars province). In French it is called Oppoponax and in Persian Jashir. Its green sticks and leaves are used in different ways, e.g., boiled in churned sour milk and yoghurt or processed vinegar. The leaf is used for gastrointestinal disorders and lacks toxicity (Chanwitheesuck et al., 2005 ▶). To the best of our knowledge, this is the first report about anti-diabetic effects of this plant.

## Material and Methods

**Chemicals and drugs**

Alloxan monohydrate and chloroform were purchased from Sigma Chemicals, Germany. Insulin NPH from Exir pharmaceutical company, normal saline from Iran Daroupakhsh company, ethanol from Pakdis company, Iran and other materials were purchased from Merk company, Germany.

**Plant material**

Fresh, green P.f plants were collected from the Shahidan Mountains of West Azerbaijan in northwest of Iran in frontier localities between Iran and Turkey in May 2010 and authenticated by a professor from the Department of Biology at Urmia University. The samples (roots separately, and green leaf and stems had weight rate of 1:1) were dried in shadow for 7 days.

**Preparation of extracts**

Collected samples were dried and ground by an electrical mill. 100 grams of both powder samples were added to 1000 ml of alcohol. First, ethanol 96% was used and after 24 h both solutions were filtered and in the second step ethanol 70% was added to the remained dry materials. After 24 h, solutions were filtered and then both filtered solutions were mixed together and then evaporated repeatedly to half the first volume by rotary evaporator in 50º C and 70 rpm. Concentrated extracts were dried on water bath at 40º C temperature to yield 6% w/w dry extract. For the preparation of injected extract, this powder was solved in specific volume of normal saline (Larkins et al., 2004 ▶).

**Preparation of diabetic rats**

Alloxan monohydrate dissolved in saline was injected to rats intra peritoneally at dose of 120 mg/kg body weight. After a fortnight, rats with marked hyperglycemia (serum glucose more than 200 mg/dl) were selected and used for the study (Kazerooni et al., 2006 ▶).

**Experimental design**

Forty female Wistar rats with BW of 200±20 g were purchased from Pasteur Institute, Iran and were kept in animal houses of Urmia University. They were kept at 20±5º C, relative humidity of 30±5% and light/dark cycle for 12h. All animals were fed with rodent pellet diet and water was allowed ad-labium under strict hygienic conditions. These rats were randomly divided into 5 groups with 8 rats per group, as follows: group 1 (C: controlled group) were administrated 0.5 ml saline, group 2 (D: untreated diabetic rats), group 3 (D+S1) diabetic rats receiving roots hydro-alcoholic extract of P.f at 100 mg/kg B.W in saline, group 4 (D+S2) diabetic rats received leaves and stems hydro-alcoholic extract of P.f at 100 mg/kg B.W in saline, group 5 (D+S3) diabetic rats received insulin NPH at 1 I.U./kg. Treatments periods were 4 weeks and all extract were given orally in rats by intra-gastric tube.

**Tissue preparation and biochemical estimation**

At the end of the experiment, the rats were weighed, anesthetized by diethyl ether and their livers were taken out and fixed in 10% natural buffer formalin. After tissue processing, the samples were blocked in cylindrical paraffin blockers and then stained by Hematoxilin- eosin (Dhandapani et al., 2002 ▶) (each sample's diameter was 5-6 microns). At first, WBC count was measured from rats' blood collected from left ventricle of the heart. Serum samples were collected for estimation of biochemical parameters from all the experimental rats including serum glucose, ALT and AST.

**Statistical analysis**

All values are expressed as Mean±SEM. The differences were compared using one way analysis of variance (ANOVA) followed by Tukey's multiple comparison tests. p-values <0.05 were considered statistically significant.

## Results

**Figure 1:**
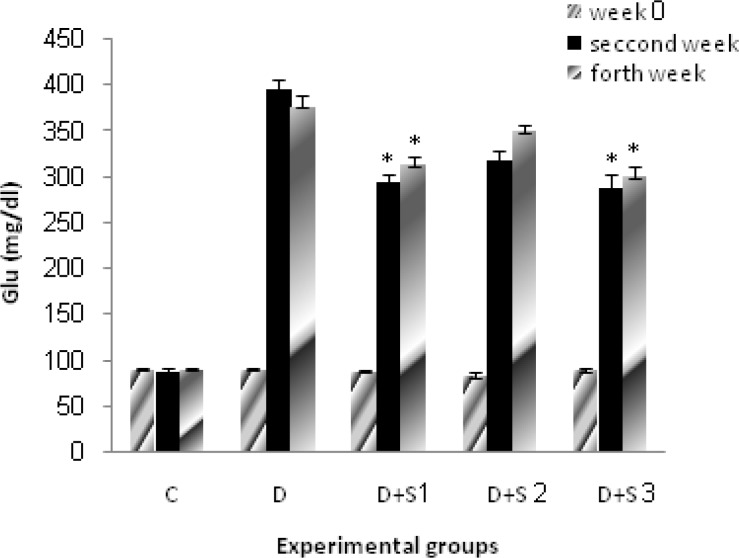
Effect of hydroalcoholic extract of P.f on serum glucose level in experimental groups on 0, 14 and 28 days. All values are expressed as Mean±SEM (n=8). Statistical comparisons between each group were carried out by one way ANOVA followed by Tukey´s multiple comparison tests.

**Figure 2:**
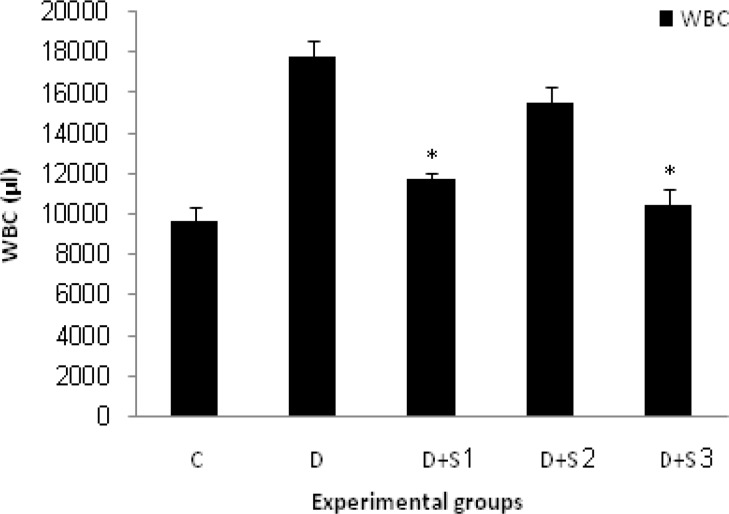
Effect of hydroalcoholic extract of P.f on WBC level in experimental groups on 28 days. All values are expressed as Mean±SEM (n=8). Statistical comparisons between each group were carried out by one way ANOVA followed by Tukey´s multiple comparison tests. *p<0.05 in comparison with diabetic values.

**Figure 3:**
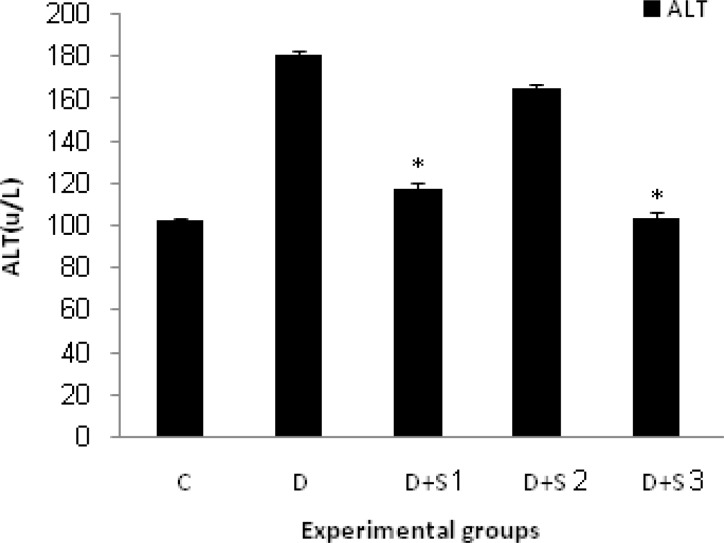
Effect of hydroalcoholic extract of P.f on serum ALT level in experimental groups on 28 days. All values are expressed as Mean± SEM (n=8). Statistical comparisons between each group were carried out by one way ANOVA followed by Tukey´s multiple comparison tests.

**Figure 4:**
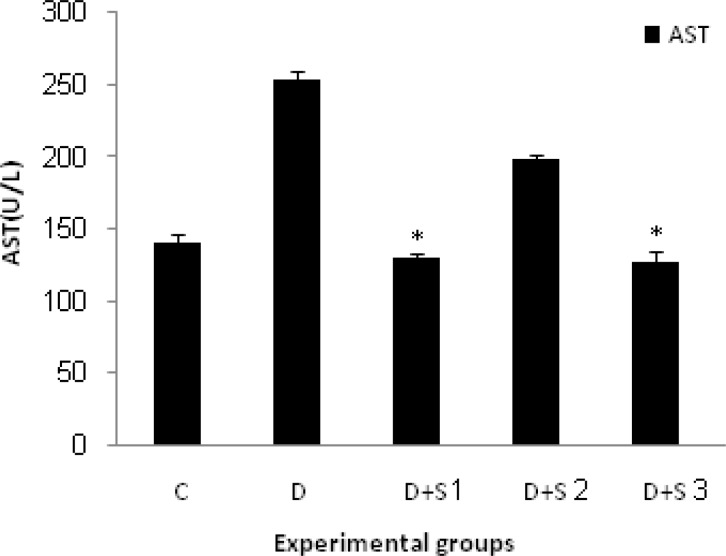
Effect of hydroalcoholic extract of P.f on serum AST level in experimental groups on 28 days. All values are expressed as Mean±SEM (n=8). Statistical comparisons between each group were carried out by one way ANOVA followed by Tukey´s multiple comparison tests.

In untreated diabetic rats, glucose, WBC, ALT and AST values were significantly elevated during the study whereas their body weight decreased. Chronic treatment with roots hydro-alcoholic extract of P.f at 100 mg/kg significantly (p<0.05) decreased WBC, serum glucose, ALT and AST as shown in Table1 and Figure 1, 2, 3 and 4.

**Table 1:** Body weight of experimental groups at the begining and the end of the experiment.

**Treatment Time**	**Control**	**Diabetic**	**D+S1**	**D+S2**	**D+S3**
**At the start of exp.**	207.4±11.3	204±10.5	210±13.2	213.1±10.8	205.6±15.3
**At the end of exp.**	254.6±21.4**	139.9±19.2	200.1±26.4**	143.2±17.2	211.4±23.4**

(**) Significant at p<0.05 as compared to diabetic group. D+S1:diabetic rats treated with roots hydroalcoholic extract of P.f (100 mg/kg), D+S2: diabetic rats treated with stems & leaves hydroalcoholic extract of P.f (100 mg/kg), D+S3:diabetic rats treated with insulin NPH (1 IU/kg)

*p<0.05 in comparison with diabetic values.

C: control group, D: diabetic group, D+S1: diabetic rats treated with roots hydroalcoholic extract of P.f (100 mg/kg), D+S2: diabetic rats treated with stems & leaves hydroalcoholic extract of P.f (100 mg/kg)

D+S3: diabetic rats treated with insulin NPH (1 IU/kg)

C: control group, D: diabetic group, D+S1: diabetic rats treated with roots hydroalcoholic extract of P.f (100 mg/kg), D+S2: diabetic rats treated with stems & leaves hydroalcoholic extract of P.f (100 mg/kg)

D+S3: diabetic rats treated with insulin NPH(1 IU/kg)

*p<0.05 in comparison with diabetic values.

C: control group, D: diabetic group, D+S1: diabetic rats treated with roots hydroalcoholic extract of P.f (100 mg/kg), D+S2: diabetic rats treated with stems & leaves hydroalcoholic extract of P.f (100 mg/kg)

D+S3: diabetic rats treated with insulin NPH(1 IU/kg)

*p<0.05 in comparison with diabetic values.

C: control group, D: diabetic group, D+S1: diabetic rats treated with roots hydroalcoholic extract of P.f (100 mg/kg), D+S2: diabetic rats treated with stems & leaves hydroalcoholic extract of P.f (100 mg/kg)

D+S3: diabetic rats treated with insulin NPH(1 IU/kg)

**Livers histo-pathological result**

**Figure 5:**
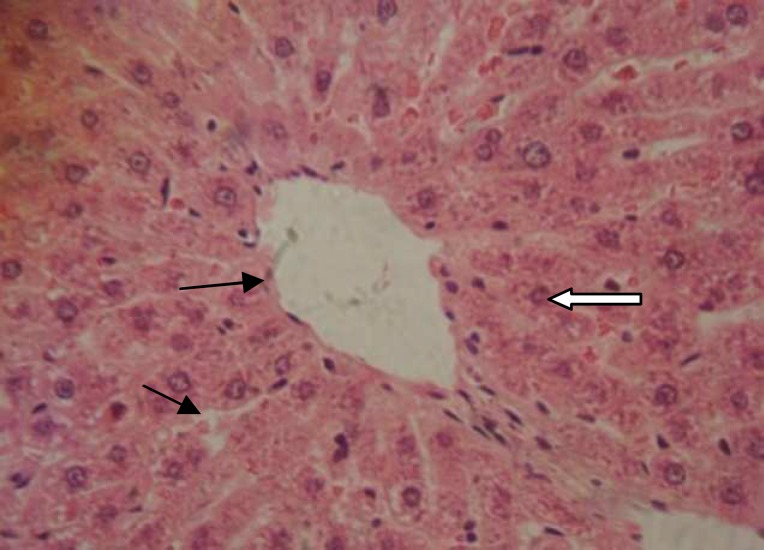
Normal rat liver (H&E×400 ) showing normal hepatocytes, sinusoides and central vein.

In the current study, in diabetic livers, an important fatty change was not observed. Normal liver of the rat is essentially formed of hepatic lobules. Each lobule is made up of radiating plates, strands of cells forming a network around a central vein with narrow sinusoids (Figure 5).

Destructive changes were more obvious in the animals of group D and D+E2. There was loss of usual concentric arrangement of hepatocytes and portal vessels and the sinusoids were congested. In group D, obvious leukocyte infiltration (Figure 6a) was observed. Further in this group, the hepatocytes appeared to suffer from certain degree of cloudy swelling with marked cytoplasmic vacuolations (Figure 6b), and nuclei of some cells revealed clear signs of pycnosis (Figure, 6b). Treatment with root hydroalcoholic extract of P.f (Figure 7) and insulin (Figure 9) showed improvement in histological structure of liver sections of diabetic rats, pronounced normalized appearance of liver lobules with strains of hepatocytes compared with the diabetic rat livers. In these groups, the hepatocytes exhibited some degree of histological regeneration, less sinusoids congestion with absence of leukocyte infiltration and less necrotic cells. In D+S2 group, most of the signs were similar to the D group and treating with plant extract has no significant effect on histopathological changes induced by alloxan injection (Figure 8).

**Figure 6 (a):**
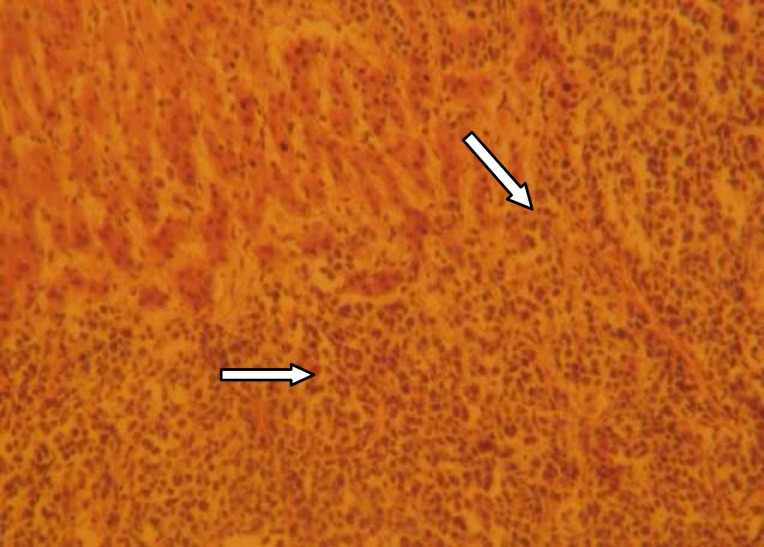
Diabetic control rats (H&E×100) showing remarkable lymphoctytic infiltration.

**Figure 6 (b):**
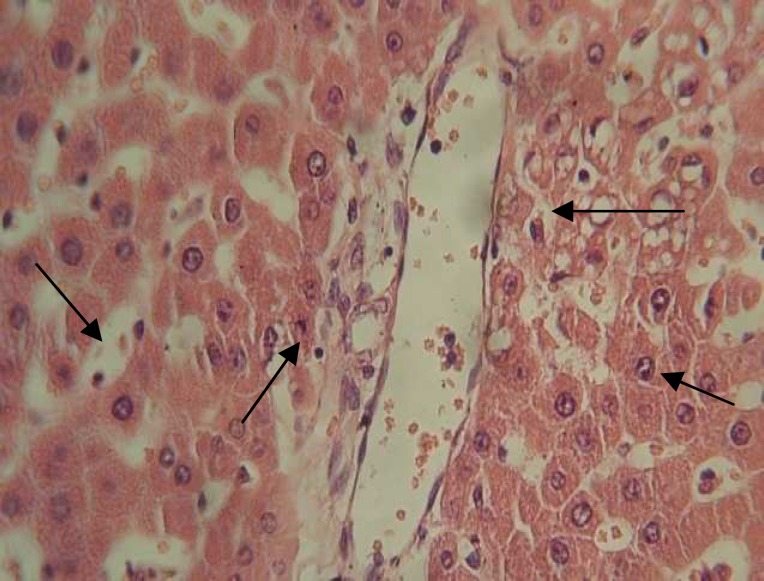
Diabetic control rats (H&E×400) showing marked sytoplasmic vacuolations, dispersed necrosis of hepatosytes with dilated sinusoids.

**Figure 7:**
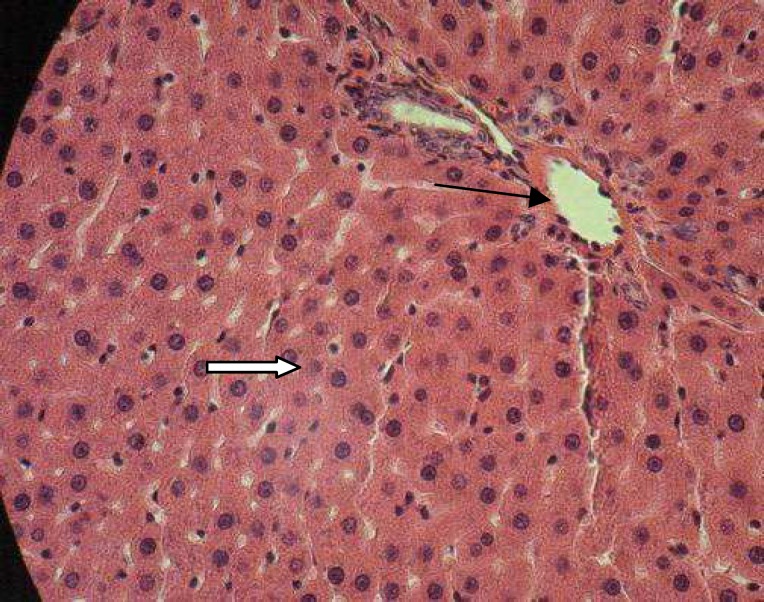
Diabetic+roots hydroalcoholic extract of P,f (100 mg/kg) treated rat liver (H&E×400) showing hypertrophic hepatocytes with normal central vein. Lymphocytic infiltrations were not observed.

**Figure 8:**
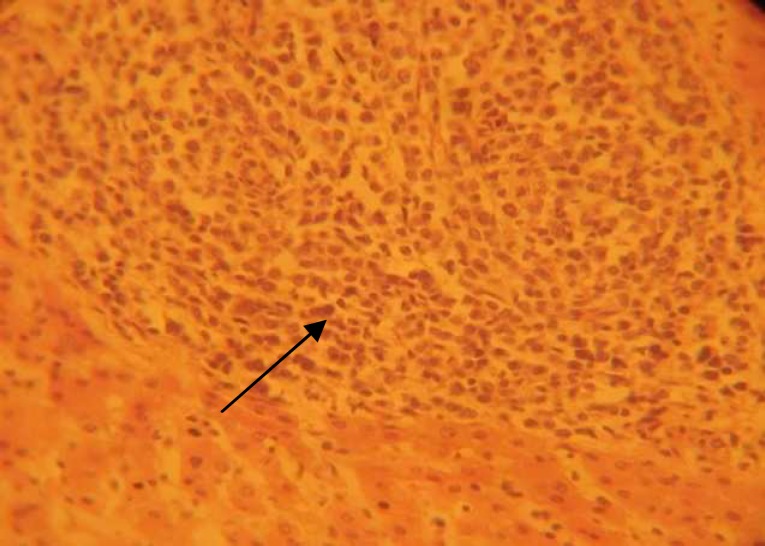
Diabetic+stems & leaves (100 mg/kg) hydroalcoholic extract of P.f treated rat liver (H&E×400) showing lymphocytic infiltration.

**Figure 9:**
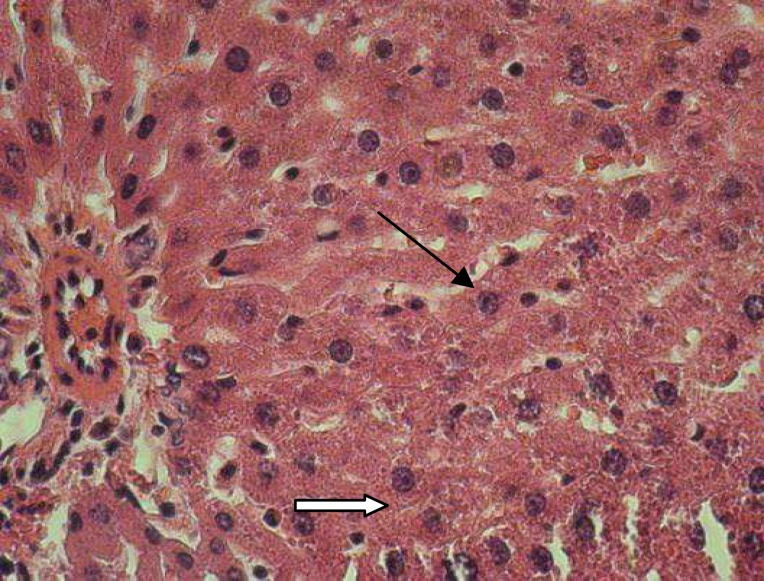
Diabetic+Insulin (1 IU/kg) treated rat liver (H&E×400) showing normal structure with hypertrophic hepatocytes and narrowed sinusoids. Lymphocytic infiltrations were not observed.

## Discussion

In this study, we investigated the effect of hydro-alcoholic extract of P.f on hepatic enzyme changes and livers histopathology during the diabetes mellitus (D.M) conditions and observed that alloxan injection caused increased serum activities of ALT and AST. In addition, in histological study of liver, obvious lymphocytic inflammation in portal areas, dilated sinusoids, and necrosis of hepatocytes were the most common hepatic injuries in D group in comparison with other groups.

D+S1 group showed a significant decrease in serum ALT and AST, and activities of both enzymes came back to the normal level in comparison with D group. In the D+S2 group, all of the signs were similar to the D group and treatment with plant extract didn’t have any significant effect on liver and hepatic enzymes. In most of the other studies, DM condition was associated with biochemical, histopathological, and physiological changes of liver (Worbozet., 2003 ▶; Pratt et al., 2009 ▶). Most importantly, recent studies showed dramatic changes of liver such as fatty liver or glycogenesis and liver structural changes in short time (Zafar et al., 2009 ▶). 

Mostly, infectious disease has been treated with herbal remedies through the history of mankind. Even today, plant materials play a major role in primary health care as therapeutic remedies in many countries. The ethno botanical information reports about 800 plants that may possess anti diabetic potential (Jones et al., 2004 ▶).

The genus of *Prangos* with the common Persian name of Jashir includes 15 species which are growing widely in many regions of Iran. Some species are distributed in Anatolia, central Asia, and Caucasian (Alarcon et al., 2008 ▶). *Prangos ferulacea (L.) *Lindle is a plant native to the mountains of southern Iran (Fars province) (Coruh et al., 2007 ▶). Previous phytochemical studies of this plant have indicated the presence of coumarines, alkaloids, flavenoides, and terpenoids. It is more important that some components such as umbelliferone, frundenole, fruliden, prangon, and penthyl coumarins were detected in the root of this plant (Alarcon et al., 2008 ▶). Plant-derived phenol coumarins might play a role as dietary antioxidants because of their consumption in the human diet especially in fruits and vegetables (Jones et al., 2004 ▶). Umbelliferon (7-hydroxy coumarin), an excellent natural antioxidant, benzopyrone in nature, is an abundant compound in roots of P.f (Coruh et al., 2007 ▶). The parent compound –coumarin- has been reported to reduce plasma glucose. Recently, Ramesh et al. reported the effect of umbliferon on glycemic control and lipids and antioxidant, glycoprotein components and hepatic marker enzymes in STZ-diabetic rats (Ramesh et al., 2005 ▶). Liver is the main organ for maintaining plasma glucose levels within narrow limits (Yamatani et al., 1994 ▶). The increase of free radical mediated toxicity is well documented in clinical diabetes and STZ or Alloxan-diabetic rats. Hyperglycemia can generate a redox imbalance inside the cells, especially in the liver (Zafar et al., 2009 ▶). Free radicals result in the consumption of antioxidant defenses which may lead to disruption of cellular function. In an experimental study in rats, a marked decrease in liver weight was observed in diabetic rats that could be due to an increased breakdown of glycogen and protein degradation and increased gluconeogenesis. In that research, treatment with umbelliferone elevated liver weight, glycogen content, and plasma proteins which can be due to increased plasma insulin level (Coruh et al., 2007 ▶). Further antioxidant property of umbelliferone may also contribute by reducing tissue damage. Enzymes directly associated with the conversion of amino acids to ketoacids are ALT and AST (Parmar et al., 1982 ▶; Khaki et al., 2009 ▶). ALT and AST activities are used as the indicators of hepatocytes damage. In earlier stage of livers damage, these cytoplasmic enzymes of hepatocytes penetrate the cells and enter the blood stream (Parmar et al., 1982 ▶; Ramesh et al., 2006 ▶). These enzymes have increased activities in diabetic rats which are due to hepatic damage and treatment while roots hydro alcoholic extract of P.f decreased the activities of these enzymes, most likely due to its antioxidant properties.

In summary, our study showed that roots hydro-alcoholic extract of P.f affects changes of aminotransferases and prevents the histopathological changes of liver in association with alloxan induced diabetes in rats and this effect can be due to the flavenoides, umbelliferone, and their antioxidant features.
